# Exercise training prevents skeletal muscle plasma membrane cholesterol accumulation, cortical actin filament loss, and insulin resistance in C57BL/6J mice fed a western‐style high‐fat diet

**DOI:** 10.14814/phy2.13363

**Published:** 2017-08-15

**Authors:** Ashley G. Ambery, Lixuan Tackett, Brent A. Penque, Joseph T. Brozinick, Jeffrey S. Elmendorf

**Affiliations:** ^1^ Department of Cellular and Integrative Physiology Indiana University School of Medicine Indianapolis Indiana; ^2^ Center for Diabetes Metabolic Disease Indiana University School of Medicine Indianapolis Indiana; ^3^ Department Biochemistry and Molecular Biology Indiana University School of Medicine Indianapolis Indiana; ^4^ Eli Lilly and Company Indianapolis Indiana

**Keywords:** Actin, cholesterol, exercise, insulin

## Abstract

Insulin action and glucose disposal are enhanced by exercise, yet the mechanisms involved remain imperfectly understood. While the causes of skeletal muscle insulin resistance also remain poorly understood, new evidence suggest excess plasma membrane (PM) cholesterol may contribute by damaging the cortical filamentous actin (F‐actin) structure essential for GLUT4 glucose transporter redistribution to the PM upon insulin stimulation. Here, we investigated whether PM cholesterol toxicity was mitigated by exercise. Male C57BL/6J mice were placed on low‐fat (LF, 10% kCal) or high‐fat (HF, 45% kCal) diets for a total of 8 weeks. During the last 3 weeks of this LF/HF diet intervention, all mice were familiarized with a treadmill for 1 week and then either sham‐exercised (0 m/min, 10% grade, 50 min) or exercised (13.5 m/min, 10% grade, 50 min) daily for 2 weeks. HF‐feeding induced a significant gain in body mass by 3 weeks. Sham or chronic exercise did not affect food consumption, water intake, or body mass gain. Prior to sham and chronic exercise, “pre‐intervention” glucose tolerance tests were performed on all animals and demonstrated that HF‐fed mice were glucose intolerant. While sham exercise did not affect glucose tolerance in the LF or HF mice, exercised mice showed an improvement in glucose tolerance. Muscle from sham‐exercised HF‐fed mice showed a significant increase in PM cholesterol, loss of cortical F‐actin, and decrease in insulin‐stimulated glucose transport compared to sham‐exercised LF‐fed mice. These HF‐fed skeletal muscle membrane/cytoskeletal abnormalities and insulin resistance were improved in exercised mice. These data reveal a new therapeutic aspect of exercise being regulation of skeletal muscle PM cholesterol homeostasis. Further studies on this mechanism of insulin resistance and the benefits of exercise on its prevention are needed.

## Introduction

Insulin resistance starts years before type 2 diabetes (T2D) diagnosis, even before recognition of prediabetes (Mason et al. 2007; Tabak et al. 2009). From a public health standpoint, physical activity is central to both the prevention and treatment of T2D, yet the exact mechanisms for this effect remain imperfectly understood. For some time it has been known that muscle contractions stimulate GLUT4‐mediated glucose uptake independent of insulin (Constable et al. 1988; Douen et al. 1990; Ryder et al. 2001). Advances in our understanding of the contraction‐stimulated signal to GLUT4 has been made through the years and presented in several recent reviews (Cartee 2015a; Rohling et al. 2016; Sylow et al. 2017). More pertinent to studies conducted in this report is that exercise increases skeletal muscle insulin sensitivity by poorly understood local changes in insulin action and GLUT4 functionality (Holloszy 2005; Maarbjerg et al. 2011; Cartee 2015b). For example, animal and human studies report that exercise increases insulin sensitivity without a simultaneous increase in proximal insulin signaling (Goodyear et al. 1995; Wojtaszewski et al. 1997, 2000; Funai et al. 2009). While a surprising finding that enhanced proximal insulin signaling is not a feature of the exercise effect, this corresponds well with an emerging appreciation that defects in proximal insulin signaling may not represent a major node of insulin resistance (Hoehn et al. 2008; Hoy et al. 2009). As enhanced skeletal muscle insulin‐stimulated glucose transport following exercise results from, at least in part, increased sarcolemmal GLUT4 content (Hansen et al. 1998; Geiger et al. 2006), a defect in GLUT4 trafficking not coupled to proximal insulin signaling must be alleviated by exercise. Importantly, this increase in insulin‐stimulated GLUT4 translocation after exercise is not dependent upon elevated muscle total GLUT4 content (Fisher et al. 2002). Notwithstanding, however, with multiple bouts of exercise muscle GLUT4 expression and content does increase and represents a key beneficial adaptation of chronic exercise along with increased capillary density, increased expression of other proteins regulating metabolism, and increased abundance of oxidative muscle fibers(Richter and Hargreaves 2013; Sylow et al. 2017). What remains elusive, however, are the mechanisms involved in the enhancement of insulin‐stimulated GLUT4‐mediated glucose transport postexercise. Several possibilities exist including a residual effect of contraction‐stimulated glucose transport. However, this insulin‐mimetic action of exercise is mostly reversed by ~2‐3 h postexercise, whereas enhanced muscle insulin sensitivity, detectable at ~1‐4 h postexercise, can persist for up to 24‐48 h (Cartee 2015b). While enhanced microvascular perfusion could also contribute, several studies have reported that an intact systemic circulation is not necessary for developing increased insulin sensitivity after exercise (Gulve et al. 1990; Funai et al. 2010; Sjoberg et al. 2017).

Novel translational findings from our laboratory now afford a fresh perspective on how caloric excess may contribute to the deterioration of insulin action, perhaps early in the development of insulin resistance. We have found that increased hexosamine biosynthesis pathway (HBP) activity in fat/muscle cells increases O‐linked N‐acetylglucosamine modification of the transcription factor Sp1, leading to transcriptional activation of HMG‐CoA reductase, the rate‐limiting enzyme in cholesterol biosynthesis (Bhonagiri et al. 2011; Habegger et al. 2012a; Penque et al. 2013). This HBP‐induced cholesterolgenic transcriptional response increases cholesterol in the plasma membrane (PM), while reducing cortical filamentous actin (F‐actin) that is essential for insulin‐stimulated GLUT4‐mediated glucose transport (Tsakiridis et al. 1994; Brozinick et al. 2004; Torok et al. 2004; Bhonagiri et al. 2011; Habegger et al. 2012a; Penque et al. 2013). Strikingly, inhibition of the HBP or Sp1 binding to DNA blocked both PM cholesterol accumulation, F‐actin loss, and GLUT4/glucose transport dysregulation (Bhonagiri et al. 2011; Habegger et al. 2012a; Penque et al. 2013). We have also shown that insulin‐stimulated glucose disposal in mice, rats, swine, and humans is inversely related to PM cholesterol content (Habegger et al. 2012a) and that normalization of PM cholesterol restores insulin responsivity (Bhonagiri et al. 2011; Habegger et al. 2012a,b). In support of PM cholesterol toxicity representing an early etiological factor of insulin resistance, key insulin signaling events (e.g., IR→IRS→PI3K→Akt2→AS160) are sufficiently intact in several in vitro and in vivo models of HBP‐induced insulin resistance (Kralik et al. 2002; Chen et al. 2003; Habegger et al. 2012a).

This PM cholesterol accumulation and insulin resistance model is in accord with recent gene expression network studies (Ding et al. 2015; Fall et al. 2015). In these large data sets, alterations in a network of coexpressed cholesterol metabolism genes were associated with T2D susceptibility. This network included genes related to cholesterol uptake, synthesis, and efflux ‐ producing a molecular profile expected to increase intracellular cholesterol. Given the lack of clarity on how skeletal muscle insulin sensitivity is improved after exercise, we tested the possibility that exercise improves membrane cholesterol and actin cytoskeletal aspects of GLUT4 regulation. Data presented support this notion.

## Materials and Methods

### Mice

Male C57BL/6J mice were obtained at 4 weeks of age from Jackson Laboratory, Bar Harbor, ME. All mice were singly housed in conventional cages and maintained on a 12‐h light/dark cycle. Body weight and food intake were recorded weekly. All animal protocols were approved by the IUSM Institutional Animal Care and Use Committee.

### Diet

Upon arrival to our facility, all mice had free access to water and standard laboratory chow for 2 wks. Following this 2‐week acclimation period, all mice received a low‐fat (LF) diet containing 20% kcal from protein, 70% kcal from carbohydrates, and 10% kcal from fat (D01030107, Research Diets Inc., New Brunswick, NJ) for 3 wks to adapt to the modified diet. This LF, as well as the high‐fat (HF), diet represented modified forms of the standard LF (D12450B) and HF (D12451) diets from Research Diets Inc., with adaptations regarding type of fat (palm oil instead of lard) and carbohydrates, to better mimic the fatty acid (FA)/carbohydrate composition of the average human diet in Western societies (de Wit et al. 2008). Following this 5‐wk acclimation/diet adaptation period, mice were either left on the LF diet or switched to the HF diet containing 20% kcal from protein, 35% kcal from carbohydrates, and 45% kcal from fat (D01030108) for 8 wks. This HF diet mimics the percent of saturated to monounsaturated to polyunsaturated FAs (40:40:20).

### Exercise

During the last 3 weeks of the LF/HF diet intervention, all mice were familiarized with a treadmill for 1 week and then either sham‐exercised (0 m/min, 10% grade, 50 min) or exercised (13.5 m/min, 10% grade, 50 min) daily for 2 weeks. Throughout the 1‐wk familiarization and 2‐wk exercise intervention, mice were maintained on either their LF‐ or HF‐diets. Following the last sham‐exercise or exercise session, mice were fasted for 5‐6 h before analyses, a time when elevated insulin‐independent glucose uptake during exercise is mostly reversed; that is, ~2‐3 h postexercise (Cartee 2015b).

### Glucose tolerance test

For the intraperitoneal glucose tolerance test, 5‐6 h fasted mice were administered glucose (2 g/kg mass i.p.). Tail vein blood glucose was measured at times indicated with an AlphaTRAK blood glucose meter (Abbott Laboratories, Inc. Alameda, CA).

### Actin analyses

A thin slice of mixed hindlimb skeletal muscle was labeled, mounted in Vectashield, and analyzed via confocal microscopy (LSM 510 NLO; Zeiss, Thornwood, NY) as previously described (Brozinick et al. 2004). Prior to imaging, all samples were de‐identified to ensure an objective analysis. All images were taken in the same focal plane of the section and under identical microscopic parameters. Images shown are representative of 5‐7 fields from each sample.

### Cholesterol analyses

Mixed hindlimb skeletal muscle crude PM pellets obtained from differential centrifugation were resuspended in 0.2 ml of HES buffer, and cholesterol content was assayed using the Amplex Red Cholesterol Assay Kit (Molecular Probes), as previously described (Bhonagiri et al. 2011).

### Measurement of glucose transport

Mice in the postprandial state were rapidly euthanized by cervical dislocation and extensor digitalis longus (EDL) muscles were dissected out, blotted on gauze, and transferred to 25 ml Erlenmeyer flasks containing 2 ml of Krebs‐Henseleit buffer (KHB) with 0.1% bovine serum albumin (BSA), 32 mM mannitol, and 8 mM glucose. The flasks were incubated in a shaking water bath maintained at 30°C for 1 h, and were continually gassed with 95% CO_2_. Muscles were initially incubated for 60 min prior to incubation under basal conditions or stimulation with submaximal insulin (60 *μ*U/ml). The muscles were then transferred to flasks containing 2 ml of KHB with 0.1% BSA, 40 mM mannitol, 2 mM pyruvate and the same additions as in the previous incubation. Glucose transport activity was measured using 2‐deoxyglucose (2‐DG) as described in detail previously (Habegger et al. 2012b).

### Statistical analyses

Values presented are means ±SEM. The significance of differences between means was evaluated by ANOVA. Where a difference was indicated by ANOVA, a Newman‐Keuls post hoc test was conducted to compare differences between groups. Statistical comparisons of the change of pre‐ and post‐exercise glucose tolerance were performed by a paired, two‐tailed Student's *t* test. Area under the curve (AUC) with respect to the increase was calculated for all GTT measures. GraphPad Prism 7 software was used for all analyses. *P*<0.05 was considered significant.

## Results

**Figure 1:**
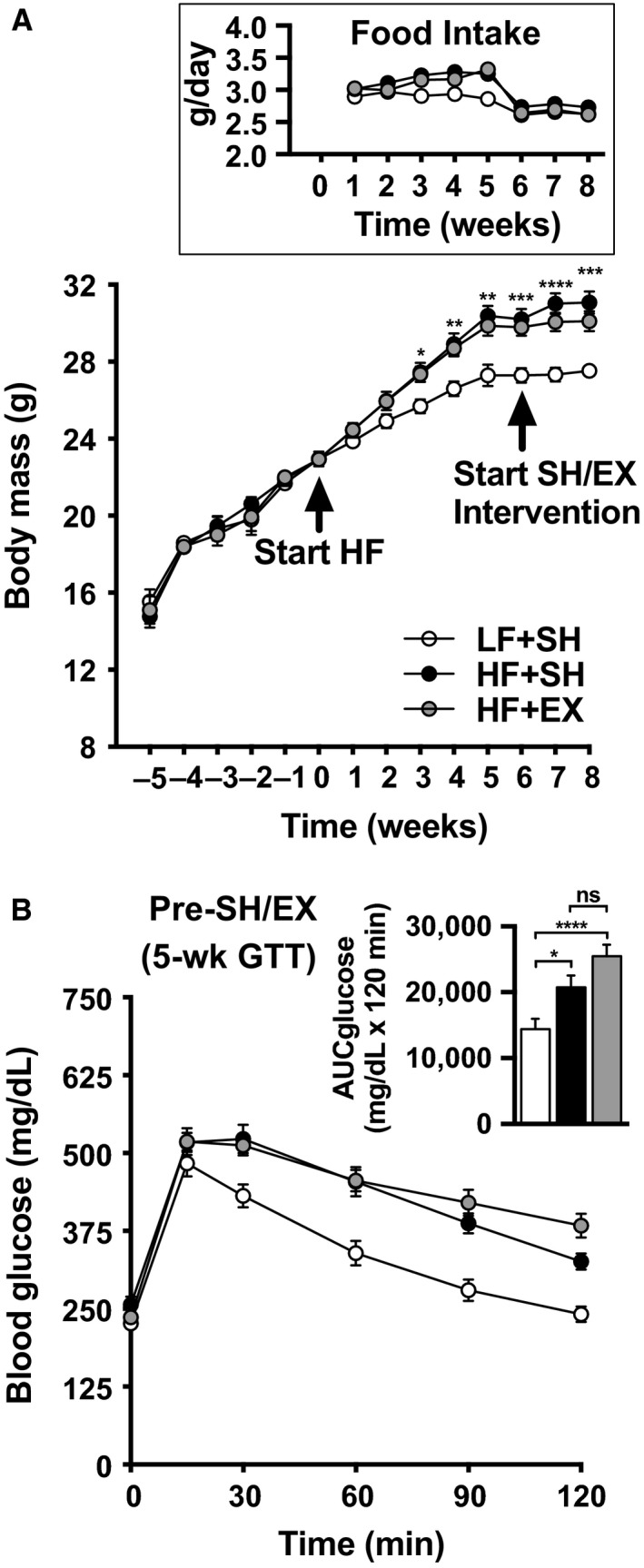
(A) Body mass during the 5‐week acclimation period (weeks ‐5 to 0), 8‐week low‐fat (LF) or high‐fat (HF) diet (weeks 0–8), and the 2‐week sham (SH) or exercise (EX) interventions (weeks 6–8). The inset shows food intake during the 8‐week diet and 2‐week SH/EX intervention. Body mass and food intake values are means ± SEM from 17 LF + SH, 18 HF + SH, and 18 HF + EX mice. (B) Pre‐SH/EX intervention glucose tolerance was determined at 5 weeks. Blood glucose was measured before and after injection of 2 g glucose/kg body mass. Glucose values are means ± SEM from 17 LF + SH, 17 HF + SH, and 17 HF + EX mice. Two‐way ANOVA (A) and one‐way ANOVA (B, inset) post hoc analysis statistics are indicated with *P < 0.05; **P < 0.01; ***P < 0.001; and ****P < 0.0001. Note some error bars are shorter than the symbols.

Mice fed a HF diet had a significant gain in body mass by 3 wks (Fig. 1A). Sham (SH) or chronic exercise (EX) did not affect food consumption (Fig 1A, inset) or water intake (data not shown) or body mass gain (Fig. 1A). Prior to sham and exercise, “pre‐intervention” GTTs were performed on all mice and demonstrated that HF‐fed mice were glucose intolerant (Fig. 1B).

**Figure 2:**
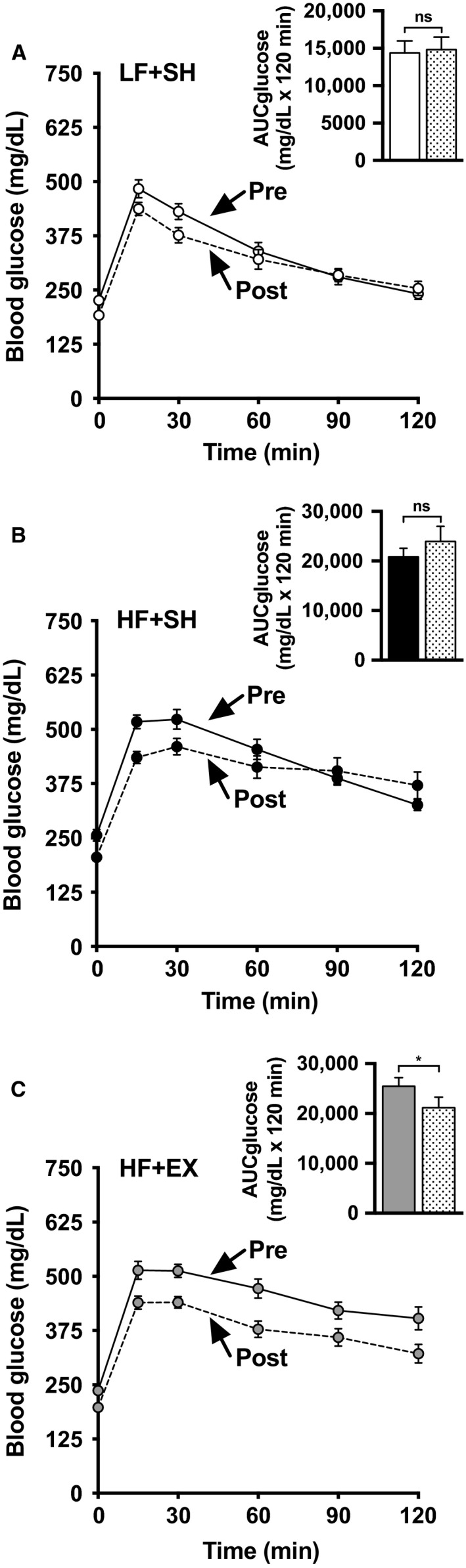
Pre‐ and post‐SH/EX intervention glucose tolerance was determined at 5‐ or 8‐ weeks, respectively, for LF‐ (A), or HF‐ (B,C) fed mice. Blood glucose was measured before and after injection of 2 g glucose/kg body mass. All values are means ± SEM from 17 LF + SH, 17 HF + SH, and 17 HF + EX mice. AUC differences were determined using a paired two‐tailed Student's *t* test and statistic indicated with ^ns^ *P *> 0.05; *P < 0.05. Solid and hatched bars represent pre‐ and post‐SH/EX interventions, respectively. Note some error bars are shorter than the symbols.

While sham exercise did not affect glucose tolerance in the LF‐ or HF‐fed mice (Figs. 2A and B, compare pre‐ and post‐tracings and solid and patterned bars), HF‐fed exercised mice showed an improvement in glucose tolerance (Fig. 2C, compare pre‐ and post‐tracings and solid and patterned bars).

**Figure 3:**
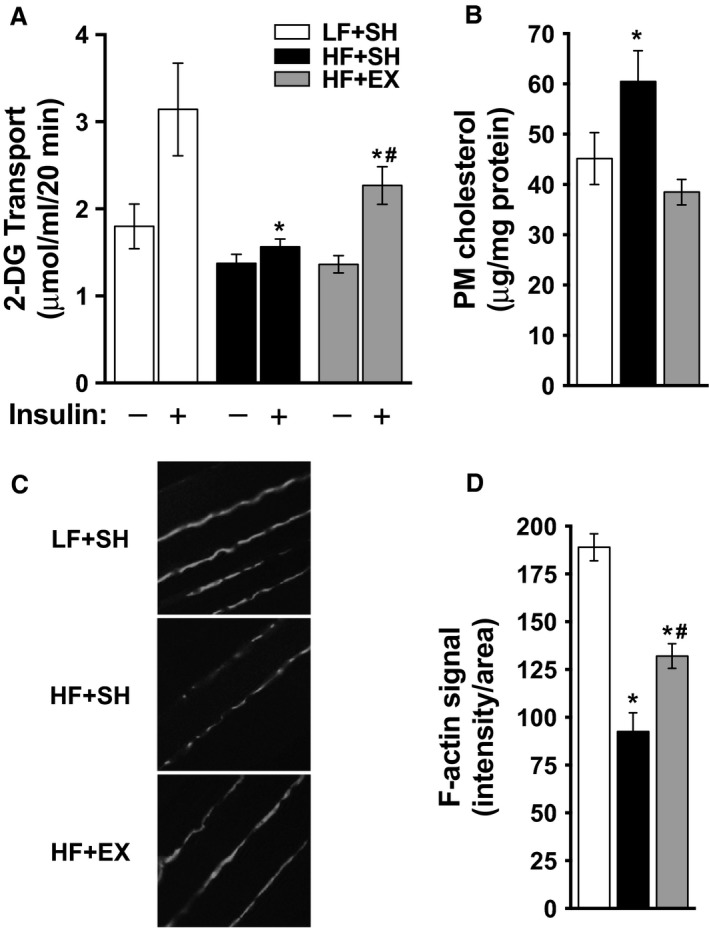
(A) Paired extensor digitalis longus muscles were subjected to basal and insulin‐stimulated 2‐DG uptake measurements as described in Methods. Uptake values are means  ± SEM from 4 LF + SH, 4 HF + SH, and 5 HF + EX muscles. (B, C, D) Mix hindlimb skeletal muscle were subjected to fractionation for membrane cholesterol determination (B) or were labeled with antibodies against F‐actin and imaged by confocal microscopy (C) and images were digitally quantitated using MetaMorph software (D) as described in Methods. Cholesterol values are means ± SEM from 12 LF + SH, 12 HF + SH, and 12 HF + EX muscles and F‐actin values are means ±SEM from 3 to 5 images of 5 LF + SH, 5 HF + SH, and 5 HF + EX muscles. One‐way ANOVA post hoc analysis statistics are indicated with *P < 0.05 (vs. LF + SH +Insulin); #*P *< 0.05 (vs. HF + SH + Insulin).

Consistent with this, muscle from sham‐exercised HF‐fed mice showed a significant decrease in insulin‐stimulated glucose transport compared to sham‐exercised LF‐fed mice (Fig. 3A, compare white and black bars). This HF‐fed‐induced decrease in insulin‐stimulated glucose transport was significantly improved, but not fully restored, in muscles from exercised mice (Fig. 3A, gray bars).

Mixed hindlimb muscle from the sham‐exercised HF‐fed mice showed a reciprocal increase in PM cholesterol (Fig. 3B, compare white and black bars) and decrease in cortical F‐actin (Figs. 3C and 3D, compare top and middle panels and white and black bars), compared to that measured in muscles from the sham‐exercised LF‐fed mice.

Exercise completely normalized the excess PM cholesterol measured in skeletal muscle from HF‐fed mice (Fig. 3B, gray bars). Similar to the measured partial improvement in insulin‐stimulated glucose transport, the HF‐fed‐induced decrease in cortical F‐actin was significantly improved, yet not fully, in muscles from exercised mice (Figs. 3C and D, bottom panel and gray bar).

## Discussion

In this work, we investigated whether excess PM cholesterol, which compromises cortical actin filaments essential for insulin‐stimulated glucose transport, is improved by exercise. We showed that muscle from sham‐exercised HF‐fed mice had increased PM cholesterol, decreased cortical F‐actin, and impaired insulin‐stimulated glucose transport compared to sham‐exercised LF‐fed mice. These observed membrane and cytoskeletal changes are recognized abnormalities that contribute to skeletal muscle insulin resistance in glucose intolerant mice, rats, swine, and humans (Habegger et al. 2012a,b; Llanos et al. 2015). Both ex vivo and in vivo exposure of insulin‐resistant skeletal muscle to methyl‐*β*‐cyclodextrin (M*β*CD) has been documented to normalize excess PM cholesterol content and improve skeletal muscle insulin sensitivity (Habegger et al. 2012b; Llanos et al. 2015). Here we similarly found that these HF‐fed skeletal muscle membrane/cytoskeletal abnormalities and insulin resistance were improved in exercised mice, and that these exercised mice showed improved glucose tolerance. While it is possible that the beneficial effects we measured could be attributed to a lingering insulin‐mimetic action of exercise, studies suggest that acute exercise‐stimulated glucose transport is mostly reversed by ~2‐3 h postexercise (Cartee 2015b). Regardless, our studies reveal that an aspect of skeletal muscle insulin resistance improved by exercise, whether acute, chronic, or a combination of both, is membrane/cytoskeletal defects that impair GLUT4 regulation by insulin. How long membrane cholesterol and/or actin filament structure remains normalized after exercise is not known. Moreover, whether the same benefit could be measured after a single bout of exercise is not known.

In a previous study, we examined the effect of increased AMP‐activated protein kinase (AMPK) activity on countering PM cholesterol accumulation and insulin resistance in L6 myotubes (Habegger et al. 2012b). An important aspect of AMPK functionality is its inactivation of energy‐consuming pathways such as fatty acid and cholesterol synthesis. We found that cells cultured in a hyperinsulinemic milieu, resembling conditions in vivo that promote the progression/worsening of insulin resistance, displayed an increase in membrane cholesterol. This occurred concomitantly with a loss of cortical F‐actin and defects in GLUT4 regulation by insulin. These derangements were prevented by AMPK stimulation. Given the well‐established effect of exercise on increasing AMPK activity, the data presented herein are consistent with the beneficial membrane/cytoskeletal effects of exercise on insulin sensitivity resulting from increased AMPK activity. We did not measure AMPK activity, as several studies have reported that AMPK activation is reversed within 30‐60 min postexercise/contraction (Rasmussen et al. 1998; Vendelbo et al. 2014). As 3‐hydroxy‐3‐methylglutaryl coenzyme A reductase (HMGR), a rate‐limiting enzyme in cholesterol synthesis, is phosphorylated and inhibited by AMPK (Clarke and Hardie 1990; Sato et al. 1993), the transient exercise‐enhanced AMPK activity is potentially important for mitigating PM cholesterol accumulation/toxicity. Also, multiple lines of evidence indicate that AMPK activation suppresses the activity of sterol‐regulatory element‐binding proteins (SREBPs) that are major transcription factors activating the expression of genes involved in biosynthesis of cholesterol, fatty acids, and triglycerides (Zhou et al. 2001; Yang et al. 2008). As such, suppression of both HMGR and SREBPs by AMPK would be predicted to lower membrane cholesterol and enhance GLUT4 regulation. In support of this possibility, inhibition of SREBP was found to ameliorate diet‐induced cholesterol/lipid tissue accumulation and insulin resistance (Tang et al. 2011).

As to why lipid lowering drugs, such as statins, are associated with increased incidence of new on‐set diabetes remains imperfectly understood, yet mechanisms have been proposed and discussed in the literature (Brault et al. 2014; Robinson 2015). Interestingly, nearly two decades of randomized, control trials and meta‐analyses have revealed that the risk is not the same among statins with atorvastatin, simvastatin, and rosuvastatin being the most diabetogenic, lovastatin and fluvastatin having an intermediate risk, and pravastatin and pitavastatin having the lowest diabetogenicity (Millan Nunez‐Cortes et al. 2016; Thakker et al. 2016). In fact, basic and clinical data suggest that these least diabetogenic statins, especially pitavastatin, may even exhibit a positive effect on glucose metabolism(Teramoto 2011; Daido et al. 2014; Huang et al. 2016). Intricately layered in our full understanding of why and how statins negatively or positively impact glycemic health is an array of factors including patient characteristics and integrative control mechanisms of cholesterol regulation. Of note in regards to skeletal muscle insulin sensitivity, is that similar to the upregulation of low‐density lipoprotein (LDL) receptors (LDLRs) that occurs in liver with cholesterol biosynthesis inhibition, muscle LDLRs and LDL cholesterol (LDL‐C) uptake are increased in mice treated with high doses of simvastatin (Yokoyama et al. 2007). It has also been found that skeletal muscle LDL‐C uptake is increased in statin‐treated mice overexpressing lipoprotein lipase (LPL) in skeletal muscle (Yokoyama et al. 2007). These data suggest that LPL [the primary enzyme for intravascular hydrolysis of triglyceride (TG)], could also be an important mediator of skeletal muscle cholesterol uptake. Notably, statins increase LPL serum mass and activity in T2D (Endo et al. 2004). Perhaps these findings offer some explanation as to why some statins increase, albeit modestly, the risk of T2D(Thakker et al. 2016). Together, these studies demonstrate evidence implicating a change in cholesterol metabolism, favoring intracellular cholesterol accumulation, as a potential risk factor for T2D. Also of interest are data suggesting statin diabetogenicity may be more coupled to defects in pancreatic *β*‐cell insulin secretory and not peripheral insulin action(Brault et al. 2014; Robinson 2015; Salunkhe et al. 2016). In accordance, Salunkhe et al. recently found dual effects on glucose homeostasis by rosuvastatin where insulin sensitivity was improved, but *β*‐cell function was impaired in high‐fat fed mice (Salunkhe et al. 2016).

Previously we demonstrated that increased hexosamine biosynthesis pathway (HBP) activity resulting from diabetic culturing conditions (e.g., hyperglycemia, hyperinsulinemia, and hyperlipidemia) increase membrane cholesterol in 3T3‐L1 adipocytes and L6 myotubes via engagement of a cholesterolgenic program (Bhonagiri et al. 2011; Habegger et al. 2012a; Penque et al. 2013). In line with increased HBP activity transcriptionally provoking a membrane cholesterol‐based insulin‐resistant state, we found that HBP inhibition attenuated the transcription and expression of genes involved in cholesterol synthesis (*e.g., Hmgcr*) and prevented membrane cholesterol accumulation, F‐actin loss, and GLUT4/glucose transport dysfunction (Bhonagiri et al. 2011; Habegger et al. 2012a; Penque et al. 2013). These data support the concept that the diabetic milieu known to accelerate diabetes progression is associated with increased de novo cholesterol synthesis. The resultant gain in skeletal muscle membrane cholesterol compromises cortical F‐actin structure that is essential for insulin‐regulated GLUT4 translocation and glucose transport. This PM cholesterol accumulation and insulin resistance model is in accord with recent gene expression network studies (Ding et al. 2015; Fall et al. 2015). In these large data sets, alterations in a network of coexpressed cholesterol metabolism genes were associated with T2D susceptibility. This network included genes related to cholesterol uptake, synthesis, and efflux ‐ producing a molecular profile expected to increase intracellular cholesterol. Data presented herein suggest for the first time that exercise may be an effective countermeasure of membrane cholesterol accumulation/toxicity.

Our analyses provide a novel new beneficial aspect of exercise, which could explain, at least in part, how exercise improves skeletal muscle insulin sensitivity. In addition to this favorable effect of exercise on mitigating PM cholesterol accumulation/toxicity, other processes underlying increased insulin sensitivity postexercise could exist. For example, several studies raise the possibility that lower glycogen and/or sustained insulin‐independent AS160 phosphorylation postexercise might sensitize skeletal muscle to insulin (reviewed in (Cartee 2015b)). While we did not measure glycogen or AS160 phosphorylation, the importance of decreased glycogen and/or increased AS160 activity toward enhanced insulin‐sensitivity is unclear (Cartee 2015b). Ex vivo and in situ analyses demonstrate that glycogen reduction and enhanced insulin sensitivity are not correlated (Cartee 2015b). However, high‐carbohydrate diet postexercise speeds glycogen resynthesis concomitant with reversal of increased insulin‐stimulated glucose uptake (Cartee 2015b). An alternative explanation could be an increased HBP cholesterolgenic response, not glycogen resynthesis, contributes to the loss of insulin sensitivity. Unlike the discordant data from in vitro and in vivo analyses where a role for glycogen is uncoupled and coupled, respectively, a role for cholesterol and cortical F‐actin is significantly coupled in both in vitro and in vivo analyses (Habegger et al. 2012a,b). With regard to a beneficial effect of exercise‐stimulated AS160 activity being favorable for subsequent insulin signal transduction, some aspects of this possibility remain unclear. For example, we previously reported that the palmitate‐induced decrease in insulin signaling in L6‐myotubes was not corrected by AMPK stimulation, yet increased PM cholesterol, reduced cortical F‐actin, and defective glucose transport in these cells were fully corrected (Habegger et al. 2012a). The observation that insulin‐stimulates a fully intact glucose transport response in cells where insulin signaling remained compromised by palmitate treatment is consistent with findings by Hoehn et al. showing that only a small percentage of total Akt activation is required for a full insulin‐stimulated glucose transport response (Hoehn et al. 2008). Together these findings suggest that an early component of insulin resistance may be membrane/cytoskeletal abnormalities that exercise, possibly via AMPK stimulation, improves. While there may exist some deficiency in insulin signaling, it is of interest that the capacity of the system may be such to still adequately transduce a sufficient signal to GLUT4 and elicit a normal redistribution of the transporter to a healthy cholesterol and cortical F‐actin cell surface environment. It is important to note, however, while we see normal insulin‐stimulated Akt2 and AS160 phosphorylation in cells displaying PM cholesterol accumulation and F‐actin loss, it is not known whether these membrane/cytoskeletal derangements could alter AS160 phosphorylation after exercise. Thus, an interesting possibility is that exercise may improve AS160 phosphorylation via mitigating membrane/cytoskeletal‐mediated suppression of AS160 phosphorylation.

In light of the recognized benefits of physical activity on reducing the incidence of T2D development in people with glucose intolerance, further study of how different types of exercise modes influence membrane and cytoskeletal aspects of insulin action are warranted. Interestingly, our exercise regime that improved insulin action was not accompanied by a concomitant loss of body mass. This observation may suggest that the effectiveness of lifestyle interventions to reduce the incidence of diabetes may not require interventions to achieve weight loss.

## Conflict of Interest

Authors have nothing to disclose.

## References

[phy213363-bib-0001] Bhonagiri, P. , G. R. Pattar , K. M. Habegger , A. M. McCarthy , L. Tackett , and J. S. Elmendorf . 2011 Evidence coupling increased hexosamine biosynthesis pathway activity to membrane cholesterol toxicity and cortical filamentous actin derangement contributing to cellular insulin resistance. Endocrinology 152:3373–3384. 21712361 10.1210/en.2011-1295 PMC3159786

[phy213363-bib-0002] Brault, M. , J. Ray , Y. H. Gomez , C. S. Mantzoros , and S. S. Daskalopoulou . 2014 Statin treatment and new‐onset diabetes: a review of proposed mechanisms. Metabolism 63:735–745. 24641882 10.1016/j.metabol.2014.02.014

[phy213363-bib-0003] Brozinick, J. T. Jr , E. D. Hawkins , A. B. Strawbridge , and J. S. Elmendorf . 2004 Disruption of cortical actin in skeletal muscle demonstrates an essential role of the cytoskeleton in glucose transporter 4 translocation in insulin‐sensitive tissues. J. Biol. Chem. 279:40699–40706. 15247264 10.1074/jbc.M402697200 PMC2409066

[phy213363-bib-0004] Cartee, G. D. 2015a Roles of TBC1D1 and TBC1D4 in insulin‐ and exercise‐stimulated glucose transport of skeletal muscle. Diabetologia 58:19–30. 25280670 10.1007/s00125-014-3395-5 PMC4258142

[phy213363-bib-0005] Cartee, G. D. 2015b Mechanisms for greater insulin‐stimulated glucose uptake in normal and insulin‐resistant skeletal muscle after acute exercise. Am. J. Physiol. Endocrinol. Metab. 309:E949–E959. 26487009 10.1152/ajpendo.00416.2015 PMC4816200

[phy213363-bib-0006] Chen, G. , P. Liu , D. C. Thurmond , and J. S. Elmendorf . 2003 Glucosamine‐induced insulin resistance is coupled to O‐linked glycosylation of Munc18c. FEBS Lett. 534:54–60. 12527361 10.1016/s0014-5793(02)03774-2

[phy213363-bib-0007] Clarke, P. R. , and D. G. Hardie . 1990 Regulation of HMG‐CoA reductase: identification of the site phosphorylated by the AMP‐activated protein kinase in vitro and in intact rat liver. EMBO J. 9:2439–2446. 2369897 10.1002/j.1460-2075.1990.tb07420.x PMC552270

[phy213363-bib-0008] Constable, S. H. , R. J. Favier , G. D. Cartee , D. A. Young , and J. O. Holloszy . 1988 Muscle glucose transport: interactions of in vitro contractions, insulin, and exercise. J. Appl. Physiol. 1985 64:2329–2332. 3136124 10.1152/jappl.1988.64.6.2329

[phy213363-bib-0009] Daido, H. , Y. Horikawa , and J. Takeda . 2014 The effects of pitavastatin on glucose metabolism in patients with type 2 diabetes with hypercholesterolemia. Diabetes Res. Clin. Pract. 106:531–537. 25458331 10.1016/j.diabres.2014.09.048

[phy213363-bib-0010] Ding, J. , L. M. Reynolds , T. Zeller , C. Muller , K. Lohman , B. J. Nicklas , et al. 2015 Alterations of a cellular cholesterol metabolism network are a molecular feature of obesity‐related type 2 diabetes and cardiovascular disease. Diabetes 64:3464–3474. 26153245 10.2337/db14-1314 PMC4587646

[phy213363-bib-0011] Douen, A. G. , T. Ramlal , S. Rastogi , P. J. Bilan , G. D. Cartee , M. Vranic , et al. 1990 Exercise induces recruitment of the “insulin‐responsive glucose transporter”. Evidence for distinct intracellular insulin‐ and exercise‐recruitable transporter pools in skeletal muscle. J. Biol. Chem. 265:13427–13430. 2199436

[phy213363-bib-0012] Endo, K. , Y. Miyashita , A. Saiki , T. Oyama , N. Koide , H. Ozaki , et al. 2004 Atorvastatin and pravastatin elevated pre‐heparin lipoprotein lipase mass of type 2 diabetes with hypercholesterolemia. J. Atheroscler. Thromb. 11:341–347. 15644588 10.5551/jat.11.341

[phy213363-bib-0013] Fall, T. , W. Xie , W. Poon , H. Yaghootkar , R. Magi , G. Consortium , et al. 2015 Using genetic variants to assess the relationship between circulating lipids and type 2 diabetes. Diabetes 64:2676–2684. 25948681 10.2337/db14-1710

[phy213363-bib-0014] Fisher, J. S. , J. Gao , D. H. Han , J. O. Holloszy , and L. A. Nolte . 2002 Activation of AMP kinase enhances sensitivity of muscle glucose transport to insulin. Am. J. Physiol. Endocrinol. Metab. 282:E18–E23. 11739078 10.1152/ajpendo.2002.282.1.E18

[phy213363-bib-0015] Funai, K. , G. G. Schweitzer , N. Sharma , M. Kanzaki , and G. D. Cartee . 2009 Increased AS160 phosphorylation, but not TBC1D1 phosphorylation, with increased postexercise insulin sensitivity in rat skeletal muscle. Am. J. Physiol. Endocrinol. Metab. 297:E242–E251. 19435856 10.1152/ajpendo.00194.2009 PMC2711658

[phy213363-bib-0016] Funai, K. , G. G. Schweitzer , C. M. Castorena , M. Kanzaki , and G. D. Cartee . 2010 In vivo exercise followed by in vitro contraction additively elevates subsequent insulin‐stimulated glucose transport by rat skeletal muscle. Am. J. Physiol. Endocrinol. Metab. 298:E999–E1010. 20179245 10.1152/ajpendo.00758.2009 PMC2867374

[phy213363-bib-0017] Geiger, P. C. , D. H. Han , D. C. Wright , and J. O. Holloszy . 2006 How muscle insulin sensitivity is regulated: testing of a hypothesis. Am. J. Physiol. Endocrinol. Metab. 291:E1258–E1263. 16835397 10.1152/ajpendo.00273.2006

[phy213363-bib-0018] Goodyear, L. J. , F. Giorgino , T. W. Balon , G. Condorelli , and R. J. Smith . 1995 Effects of contractile activity on tyrosine phosphoproteins and PI 3‐kinase activity in rat skeletal muscle. Am. J. Physiol. 268:E987–E995. 7762655 10.1152/ajpendo.1995.268.5.E987

[phy213363-bib-0019] Gulve, E. A. , G. D. Cartee , J. R. Zierath , V. M. Corpus , and J. O. Holloszy . 1990 Reversal of enhanced muscle glucose transport after exercise: roles of insulin and glucose. Am. J. Physiol. 259:E685–E691. 2240207 10.1152/ajpendo.1990.259.5.E685

[phy213363-bib-0020] Habegger, K. M. , B. A. Penque , W. Sealls , L. Tackett , L. N. Bell , E. K. Blue , et al. 2012a Fat‐induced membrane cholesterol accrual provokes cortical filamentous actin destabilisation and glucose transport dysfunction in skeletal muscle. Diabetologia 55:457–467. 22002007 10.1007/s00125-011-2334-y PMC3245823

[phy213363-bib-0021] Habegger, K. M. , N. J. Hoffman , C. M. Ridenour , J. T. Brozinick , and J. S. Elmendorf . 2012b AMPK enhances insulin‐stimulated GLUT4 regulation via lowering membrane cholesterol. Endocrinology 153:2130–2141. 22434076 10.1210/en.2011-2099 PMC3339638

[phy213363-bib-0022] Hansen, P. A. , L. A. Nolte , M. M. Chen , and J. O. Holloszy . 1998 Increased GLUT‐4 translocation mediates enhanced insulin sensitivity of muscle glucose transport after exercise. J Appl Physiol 1985 85:1218–1222. 9760308 10.1152/jappl.1998.85.4.1218

[phy213363-bib-0023] Hoehn, K. L. , C. Hohnen‐Behrens , A. Cederberg , L. E. Wu , N. Turner , T. Yuasa , et al. 2008 IRS1‐independent defects define major nodes of insulin resistance. Cell Metab. 7:421–433. 18460333 10.1016/j.cmet.2008.04.005 PMC2443409

[phy213363-bib-0024] Holloszy, J. O. 2005 Exercise‐induced increase in muscle insulin sensitivity. J. Appl. Physiol. 1985 99:338–343. 16036907 10.1152/japplphysiol.00123.2005

[phy213363-bib-0025] Hoy, A. J. , A. E. Brandon , N. Turner , M. J. Watt , C. R. Bruce , G. J. Cooney , et al. 2009 Lipid and insulin infusion‐induced skeletal muscle insulin resistance is likely due to metabolic feedback and not changes in IRS‐1, Akt, or AS160 phosphorylation. Am. J. Physiol. Endocrinol. Metab. 297:E67–E75. 19366875 10.1152/ajpendo.90945.2008 PMC2711668

[phy213363-bib-0026] Huang, C. H. , Y. Y. Huang , and B. R. Hsu . 2016 Pitavastatin improves glycated hemoglobin in patients with poorly controlled type 2 diabetes. J. Diabetes Invest. 7:769–776. 10.1111/jdi.12483 PMC5009141 27181110

[phy213363-bib-0027] Kralik, S. F. , P. Liu , B. J. Leffler , and J. S. Elmendorf . 2002 Ceramide and glucosamine antagonism of alternate signaling pathways regulating insulin‐ and osmotic shock‐induced glucose transporter 4 translocation. Endocrinology 143:37–46. 11751589 10.1210/endo.143.1.8606

[phy213363-bib-0028] Llanos, P. , A. Contreras‐Ferrat , T. Georgiev , C. Osorio‐Fuentealba , A. Espinosa , J. Hidalgo , et al. 2015 The cholesterol‐lowering agent methyl‐beta‐cyclodextrin promotes glucose uptake via GLUT4 in adult muscle fibers and reduces insulin resistance in obese mice. Am. J. Physiol. Endocrinol. Metab. 308:E294–E305. 25491723 10.1152/ajpendo.00189.2014

[phy213363-bib-0029] Maarbjerg, S. J. , L. Sylow , and E. A. Richter . 2011 Current understanding of increased insulin sensitivity after exercise ‐ emerging candidates. Acta Physiol. (Oxf) 202:323–335. 21352505 10.1111/j.1748-1716.2011.02267.x

[phy213363-bib-0030] Mason, C. C. , R. L. Hanson , and W. C. Knowler . 2007 Progression to type 2 diabetes characterized by moderate then rapid glucose increases. Diabetes 56:2054–2061. 17473220 10.2337/db07-0053

[phy213363-bib-0031] Millan Nunez‐Cortes, J. , A. Cases Amenos , J. F. Ascaso Gimilio , V. Barrios Alonso , V. Pascual Fuster , J. C. Pedro‐Botet Montoya , et al. 2016 Consensus on the statin of choice in patients with impaired glucose metabolism: results of the DIANA Study. Am. J. Cardiovasc. Drugs 17:135–142. 10.1007/s40256-016-0197-9 PMC5340834 27837448

[phy213363-bib-0032] Penque, B. A. , A. M. Hoggatt , B. P. Herring , and J. S. Elmendorf . 2013 Hexosamine biosynthesis impairs insulin action via a cholesterolgenic response. Mol. Endocrinol. 27:536–547. 23315940 10.1210/me.2012-1213 PMC3589672

[phy213363-bib-0033] Rasmussen, B. B. , C. R. Hancock , and W. W. Winder . 1998 Postexercise recovery of skeletal muscle malonyl‐CoA, acetyl‐CoA carboxylase, and AMP‐activated protein kinase. J. Appl. Physiol. 1985 85: 1629–1634. 9804562 10.1152/jappl.1998.85.5.1629

[phy213363-bib-0034] Richter, E. A. , and M. Hargreaves . 2013 Exercise, GLUT4, and skeletal muscle glucose uptake. Physiol. Rev. 93:993–1017. 23899560 10.1152/physrev.00038.2012

[phy213363-bib-0035] Robinson, J. G. 2015 Statins and diabetes risk: how real is it and what are the mechanisms? Curr. Opin. Lipidol. 26:228–235. 25887679 10.1097/MOL.0000000000000172

[phy213363-bib-0036] Rohling, M. , C. Herder , T. Stemper , and K. Mussig . 2016 Influence of acute and chronic exercise on glucose uptake. J. Diabetes Res. 2016:2868652. 27069930 10.1155/2016/2868652 PMC4812462

[phy213363-bib-0037] Ryder, J. W. , A. V. Chibalin , and J. R. Zierath . 2001 Intracellular mechanisms underlying increases in glucose uptake in response to insulin or exercise in skeletal muscle. Acta Physiol. Scand. 171:249–257. 11412137 10.1046/j.1365-201x.2001.00827.x

[phy213363-bib-0038] Salunkhe, V. A. , I. G. Mollet , J. K. Ofori , H. A. Malm , J. L. Esguerra , T. M. Reinbothe , et al. 2016 Dual effect of rosuvastatin on glucose homeostasis through improved insulin sensitivity and reduced insulin secretion. EBioMedicine 10:185–194. 27453321 10.1016/j.ebiom.2016.07.007 PMC5006666

[phy213363-bib-0039] Sato, R. , J. L. Goldstein , and M. S. Brown . 1993 Replacement of serine‐871 of hamster 3‐hydroxy‐3‐methylglutaryl‐CoA reductase prevents phosphorylation by AMP‐activated kinase and blocks inhibition of sterol synthesis induced by ATP depletion. Proc. Natl Acad. Sci. USA 90:9261–9265. 8415689 10.1073/pnas.90.20.9261 PMC47547

[phy213363-bib-0040] Sjoberg, K. A. , C. Frosig , R. Kjobsted , L. Sylow , M. Kleinert , A. C. Betik , et al. 2017 Exercise increases human skeletal muscle insulin sensitivity via coordinated increases in microvascular perfusion and molecular signaling. Diabetes 66:1501–1510. 28292969 10.2337/db16-1327

[phy213363-bib-0041] Sylow, L. , M. Kleinert , E. A. Richter , and T. E. Jensen . 2017 Exercise‐stimulated glucose uptake ‐ regulation and implications for glycaemic control. Nat. Rev. Endocrinol. 13:133–148. 27739515 10.1038/nrendo.2016.162

[phy213363-bib-0042] Tabak, A. G. , M. Jokela , T. N. Akbaraly , E. J. Brunner , M. Kivimaki , and D. R. Witte . 2009 Trajectories of glycaemia, insulin sensitivity, and insulin secretion before diagnosis of type 2 diabetes: an analysis from the Whitehall II study. Lancet 373:2215–2221. 19515410 10.1016/S0140-6736(09)60619-X PMC2726723

[phy213363-bib-0043] Tang, J. J. , J. G. Li , W. Qi , W. W. Qiu , P. S. Li , B. L. Li , et al. 2011 Inhibition of SREBP by a small molecule, betulin, improves hyperlipidemia and insulin resistance and reduces atherosclerotic plaques. Cell Metab. 13:44–56. 21195348 10.1016/j.cmet.2010.12.004

[phy213363-bib-0044] Teramoto, T. 2011 Pitavastatin: clinical effects from the LIVES study. Atheroscler. Suppl. 12:285–288. 22152283 10.1016/S1567-5688(11)70888-1

[phy213363-bib-0045] Thakker, D. , S. Nair , A. Pagada , V. Jamdade , and A. Malik . 2016 Statin use and the risk of developing diabetes: a network meta‐analysis. Pharmacoepidemiol. Drug Saf. 25:1131–1149. 27277934 10.1002/pds.4020

[phy213363-bib-0046] Torok, D. , N. Patel , L. Jebailey , F. S. Thong , V. K. Randhawa , A. Klip , et al. 2004 Insulin but not PDGF relies on actin remodeling and on VAMP2 for GLUT4 translocation in myoblasts. J. Cell Sci. 117:5447–5455. 15466888 10.1242/jcs.01421

[phy213363-bib-0047] Tsakiridis, T. , M. Vranic , and A. Klip . 1994 Disassembly of the actin network inhibits insulin‐dependent stimulation of glucose transport and prevents recruitment of glucose transporters to the plasma membrane. J. Biol. Chem. 269:29934–29942. 7961991

[phy213363-bib-0048] Vendelbo, M. H. , A. B. Moller , J. T. Treebak , L. C. Gormsen , L. J. Goodyear , J. F. Wojtaszewski , et al. 2014 Sustained AS160 and TBC1D1 phosphorylations in human skeletal muscle 30 min after a single bout of exercise. J. Appl. Physiol. 1985 117:289–296. 24876356 10.1152/japplphysiol.00044.2014 PMC4971896

[phy213363-bib-0049] de Wit, N. J. , H. Bosch‐Vermeulen , P. J. de Groot , G. J. Hooiveld , M. M. Bromhaar , J. Jansen , et al. 2008 The role of the small intestine in the development of dietary fat‐induced obesity and insulin resistance in C57BL/6J mice. BMC Med. Genomics 1:14. 18457598 10.1186/1755-8794-1-14 PMC2396659

[phy213363-bib-0050] Wojtaszewski, J. F. , B. F. Hansen , B. Kiens , and E. A. Richter . 1997 Insulin signaling in human skeletal muscle: time course and effect of exercise. Diabetes 46:1775–1781. 9356025 10.2337/diab.46.11.1775

[phy213363-bib-0051] Wojtaszewski, J. F. , B. F. Hansen , B. F. Hansen , Gade , B. Kiens , J. F. Markuns , et al. 2000 Insulin signaling and insulin sensitivity after exercise in human skeletal muscle. Diabetes 49:325–331. 10868952 10.2337/diabetes.49.3.325

[phy213363-bib-0052] Yang, J. , S. Maika , L. Craddock , J. A. King , and Z. M. Liu . 2008 Chronic activation of AMP‐activated protein kinase‐alpha1 in liver leads to decreased adiposity in mice. Biochem. Biophys. Res. Comm. 370:248–253. 18381066 10.1016/j.bbrc.2008.03.094

[phy213363-bib-0053] Yokoyama, M. , T. Seo , T. Park , H. Yagyu , Y. Hu , N. H. Son , et al. 2007 Effects of lipoprotein lipase and statins on cholesterol uptake into heart and skeletal muscle. J. Lipid Res. 48:646–655. 17189607 10.1194/jlr.M600301-JLR200

[phy213363-bib-0054] Zhou, G. , R. Myers , Y. Li , Y. Chen , X. Shen , J. Fenyk‐Melody , et al. 2001 Role of AMP‐activated protein kinase in mechanism of metformin action. J. Clin. Invest. 108:1167–1174. 11602624 10.1172/JCI13505 PMC209533

